# First line nurse managers’ experiences of opportunities and obstacles to support evidence‐based nursing

**DOI:** 10.1002/nop2.172

**Published:** 2018-06-26

**Authors:** Malin Karlberg Traav, Henrietta Forsman, Mats Eriksson, Agneta Cronqvist

**Affiliations:** ^1^ Department of Health Care Science Ersta Sköndal Bräcke University College Stockholm Sweden; ^2^ Faculty of Medicine and Health, School of Health Sciences Örebro University Örebro Sweden; ^3^ School of Education, Health and Social Studies Dalarna University Falun Sweden

**Keywords:** coherent strategy, evidence‐based nursing, first line nurse manager, leadership, phenomenography, reflection

## Abstract

**Aim:**

The aim was to explore first line nurse managers’ experiences of opportunities and obstacles to support evidence‐based nursing.

**Design:**

A qualitative study with a phenomenographical approach.

**Method:**

Data were collected through focus group interviews with 15 first line nurse managers’ in four settings.

**Results:**

The results are presented in four categories of description headed: *Manage the everyday work* vs.* evidence‐based nursing; Uncertainties about evidence‐based nursing and nursing research; Time as a reality, as an approach*; and *Shaping awareness—towards an active approach to evidence‐based nursing*. The overarching category of description has been formulated as follows: *The internal relation—how active leadership influences evidence‐based nursing*. The outcome space is presented as: *The individual path—how to make vision and reality become a working entity around evidence‐based nursing*.

## 1 INTRODUCTION

The patients of today expect nurses to work evidence‐based in nursing (Scott & McSherry, 2009), allowing the patients to receive and experience high quality of the nursing care. Although research has found that nurses want to work in an evidence‐based way, it has been reported that nurses find it hard (Andre, Aune, & Braend, 2016; Mortenius et al., 2013; Strandberg et al., 2014) One of the most important factors in supporting nurses to work evidence‐based is nursing leadership. The first line nurse manager (FLNM) has a complex and constantly changing work situation (Skytt et al, 2015). The importance of evidence‐based nursing and how the FLNM’ needs to support the nurses concerning this, is not very well known, so it is of importance to understand how first line nurse managers’ (FLNMs’) perceive their opportunities and obstacles regarding this.

### 1.1 Background

The importance of leadership when organizing the successful implementation of evidence‐based nursing has been highlighted in numerous studies (Aarons, Ehrhart, & Farahnak, 2014; Ehrenberg, Gustavsson, Wallin, Bostrom, & Rudman, 2016; Perreira & Berta, 2016). The FLNMs’ need to have an understanding of their own importance in this process and, further, have to have the ability to support research use among nurses, for example; otherwise the nursing care will not likely be evidence‐based (Bohman, Ericsson, & Borglin, 2013; Perreira & Berta, 2016; Sandström, Borglin, Nilsson, & Willman, 2011). Research use, as an example of an activity in evidence‐based nursing, can become a part of the working culture on the ward, if the FLNM leads the clinical work in that direction (Karlberg Traav, Gabrielsson, & Cronqvist, 2014). The assignment for FLNMs’ of today is complex and their workday is filled with tasks like meetings, scheduling and organisational issues (Ericsson & Augustinsson, 2015), tasks that can hinder them from supporting evidence‐based nursing. There is a notable difference between evidence‐based nursing and evidence‐based practice. Evidence‐based nursing as seen by, for example, Scott and McSherry (2009) added nursing theory as a part of the concept. Evidence‐based practice is a multi‐professional approach towards patients, including nurses work as well as other caring professions (Scott & McSherry, 2009). First line nurse managers come from different academic backgrounds and have varying degrees of leadership training; both academic and leadership training are important prerequisites for a leadership that can support evidence‐based nursing (Clement‐O'Brien, Polit, & Fitzpatrick, 2011; Merrill, 2015; Sandström et al., 2011). The FLNM position has traditionally been based on clinical experience rather than academic merit. What was previously a role including both clinical work and supervision of nurses is today pure management (Ericsson & Augustinsson, 2015). The FLNMs’ are a function just above the nurses, with FLNMs in turn reporting to management above themselves (Skytt et al, 2015). This can cause situations of conflict for the FLNM, for example, if production is the priority of management (Ericsson & Augustinsson, 2015). So far, however, not many studies have been conducted about how FLNMs’ consider their responsibility of creating evidence‐based nursing or how to create a working climate that supports evidence‐based nursing. Therefore, it is import to explore the qualitative variations concerning FLNMs’ own conceptions and experiences of how they manage their responsibility concerning evidence‐based nursing.

### 1.2 Aim

The aim was to explore first line nurse managers’ experiences of opportunities and obstacles to support evidence‐based nursing.

## 2 METHODS

### 2.1 Study design

The study used a qualitative approach and the method used was phenomenography; therefore the focus of the study lay in the discovery of the qualitative variations in experiences, conceptions and understandings of the phenomenon in interest (Marton, 1981; Marton & Booth, 2009). Phenomenography was chosen with the goal of describing different ways of how FLNMs’ understand, “make sense” and experience their opportunities and obstacles to support evidence‐based nursing as described by Barnard, McCosker & Gerber (1999). The collected data can be understood on two levels: first order perspective can be understood as the researchers understanding of how the phenomenon in interest appears to be or “really is” (Sjöström & Dahlgren, 2002). The second order perspective is when the researcher tries to understand how the phenomenon in interest is conceived from the qualitative variations (Sjöström & Dahlgren, 2002). The outcome space will describe the internal relations between the descriptions of categories, formed from the conceptions and can be viewed as an explanatory and comprehensive presentation of the study results (C‐Y Han, Barnard, & Chapman, 2009).

### 2.2 Study context and participants

The setting for the study was a university hospital with over 30 advanced care departments. The total number of beds at the hospital was around 550. The FLNM's and/or their assistant nurse managers were invited to participate in the study. The participants had been in their current position for 1–26 years. All participants were registered nurses and their experience of being a nurse ranged from 9 to 36 years. Altogether 15 participated, 13 women and 2 men.

### 2.3 Data collection

An invitation to participate in a focus group interview was sent out by e‐mail to all FLNM's at the hospital (McLafferty, 2004), 96 individuals at the time. The invitation was supplemented with telephone calls. Altogether four focus group interviews were completed, with three to six participants in each session and a total of 15 participants. The first three focus group interviews were conducted by two of the authors, A.C. and M.K.T. and the last one by M.K.T. The interviews took place during office hours in a conference room in the hospital.

To minimize possible negative influences of the participants preunderstanding and avoiding criticism towards their perceived leadership function, we have chosen to use the results from a relevant scientific article (Lindberg, Persson, & Bondas, 2012). That study had a qualitative design aiming to explore insights into how nurses, senior preceptors and head nurses experience the integration of caring science in practice and how they value the contributions of nursing students to the integration of caring science in practice (Lindberg et al., 2012). The themes identified in that study were: (a) integration—someone else's responsibility; (b) the hospital—a culture of production; and (c) the hospital and the university—different realities. On the basis of these themes, we asked our participants: Do you recognize this? This was followed up with supplementary questions, for example: could you explain? Could you describe an episode?

### 2.4 Data analysis

The interviews were audio‐recorded and transcribed verbatim. The data were read through repeatedly to give an overall picture (familiarization). Preliminary conceptions were identified and then discussed in the research group to reach agreement on the final formulation of conceptions (articulation, condensation). Four categories of description were formulated from the conceptions and were arranged hierarchically (grouping, comparison) with the overarching category as the highest level and the outcome space representing the relationships between the conceptions (labelling, contrasting) (Chin‐Yen Han et al., 2017). The research group worked both individually and together when performing the analysis, reading and rereading the material and confirming that it was understood the same way on the second and third reading, working with a dialogical check (Collier‐Reed, Ingerman, & Berglund, 2009).

Ethical permission for the study was granted by the Regional Ethical Review Board in Uppsala (Reg. No. 2014/266). The study was conducted in line with the ethical statements made in the Declaration of Helsinki (2008). Participants were given oral and written information about the study. Voluntary participation was stressed, as was the possibility to withdraw from the study at any time without having to give a reason. Participants were assured of confidentiality and anonymity.

## 3 RESULTS

**Figure 1:**
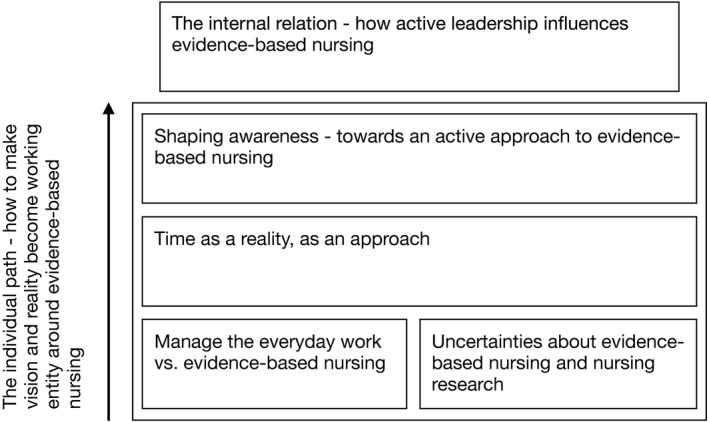
The outcome space

In the result, obstacles and opportunities could be understood in different ways. The overarching category: *The internal relation—how active leadership influences evidence‐based nursing,*views the inner journey the FLNM has to form to be able to realize how one self can be the obstacle and also how one self with insight, can be the opportunity to become supportive towards evidence‐based nursing. In the different steps shown in the Figure 1, the insight can be followed.

The results are further presented in four categories of description, under the headings: *Manage the everyday work*vs.* evidence‐based nursing; Uncertainties about evidence‐based nursing and nursing research; Time as a reality, as an approach;*and *Shaping awareness—towards an active approach to evidence‐based nursing.*Those categories showed opportunities and obstacles in relation to the clarity the participants discussed around evidence‐based nursing and their own possibility to be supportive.

The overarching category of description was worded as follows: *The internal relation—how active leadership influences evidence‐based nursing.*Finally, the outcome space was formulated as: *The individual path—how to make vision and reality become a working entity around evidence‐based nursing.*

### 3.1 Manage the everyday work vs. evidence‐based nursing

During the interviews, the participants did not clearly identify themselves as the person in charge of evidence‐based nursing. Their everyday workload got in the way, which often caused frustration. The participants felt that someone else, or an external function, should be handling how to achieve evidence‐based nursing in the clinical work at the ward. They also expressed that achievement of evidence‐based nursing was everybody's responsibility or even the hospital managements:

… because I feel that it would need a person, a special person with that knowledge, to be able to handle those questions in the group, it's got to be an enthusiast and as a first line manager you can be really supportive, but you have all the other questions and tasks to take care of, so I really think someone else should be responsible for this. ( Interview 1)

Yes, I think you are the FLNM, but still you are not and I think they [the hospital management] should change as well. I don't think we have a clear mandate in the hospital. (Interview 1)

To summarize, the participants expressed a rather confused or frustrated approach towards their own ambition and possibility to support evidence‐based nursing; that is, they conceived the obstacle of being busy. The conception that evidence‐based nursing should be someone else's or, alternatively, everybody's or even the hospital managements’ responsibility was expressed.

### 3.2 Uncertainties about evidence‐based nursing and nursing research

This category describes the participants’ way of approaching evidence‐based nursing from their point of view as FLNM's. What constituted research was described as unclear and imprecise. The participants themselves were vague and imprecise when talking about research use, for example. Nursing theory was not mentioned at all. Most of the participants said that they were aware of medical research being conducted and that the hospital had a strong medical research tradition:

… because my feeling is that the nurses, they do not, in my ward no‐one even has a thought of doing research, just doing some nursing research; everything is based on the medical tradition when it comes to research in my ward. ( Interview 3)

The participants talked about the working climate at the hospital as being focused on production and how that climate affected the way nurses thought and acted concerning evidence‐based nursing. For example, one participant expressed it this way:

Yes, at the hospital as in the culture dominated by production, you more or less get forced into it …. (Interview 1)

In this category, some of the participants expressed frustration and confusion about evidence‐based nursing, nursing research and accordingly their responsibility regarding implementation of the same. The participants conceived that it was other assignments during work hours that needed to be addressed before they could even start thinking about supporting evidence‐based nursing and that they never found themselves able to tackle the task. This frustration about the working climate was described as an obstacle.

### 3.3 Time as a reality, as an approach

The regret “if I only had more time” was frequently expressed in the focus group interviews. The participants repeatedly reported lack of time as an obstacle to be supportive to nurses when nurses wanted to use nursing research or nursing theories to improve the care. The participants seemed never get to the point where the possibility to provide support for evidence‐based nursing showed up. Their descriptions showed, however, that evidence‐based nursing was not their top priority. This was expressed as follows:

… I never end up there [supporting evidence‐based nursing, authors remark] because there is always so much work that I must do first; I put out the fires, I'm already planning the staffing for the summer and thinking how will we fix the staffing for Christmas.... (Interview 2)

…but right now, it is as it is, that you cannot stand or there is no time for anything except to try to get it all to go around at the ward… (Interview 1)

Yes, there is a lack of time due to the producing culture. (Interview 3)

This category highlights the important matter of time in health care. Lack of time is a reality; and this category is a way of looking at the FLNMs’ handling of time, which determined their ability to support the nurses to work in an evidence‐based way. The participants were aware of the importance of time, some of them in a more dejected way than others.

### 3.4 Shaping awareness—towards an active approach to evidence‐based nursing

This category is about starting a process towards an active approach to evidence‐based nursing. It is not always the FLNM know and formulate their own importance, but it is still obvious that an awareness is growing, as an opportunity. It was expressed like this:

When a question comes up, we think, well do we handle this right? Can we check this up and then we do that and you try to deal with the question the same afternoon when the nurses have their coffee break, we just take 45 min and discuss, we don't make a big fuss of it. (Interview 4)

The FLNM's understood the importance and benefit of having student nurses at the ward:

Students often have a critical approach I think, or some of them. Of course, they can highlight that there have emerged for example new routines … they can ask, “Why do you do this the way you do?” and we can start to think “Yes, why do we do it that way?” That can result in for example new routines, so I think the students are important at the ward. ( Interview 2)

The last category shows that insight is starting to take hold. Here, the participants expressed their views on evidence‐based nursing in a more active way. They sometimes downplayed their stand, but it was evident that it was important to them.

### 3.5 The overarching category: The internal relation—how active leadership influences evidence‐based nursing

The overarching category of description gives a picture of an internal relation between reflection and an active approach to support evidence‐based nursing. When the leader takes the initiative to create a climate that supports evidence‐based nursing, then the process that influences thinking, attitude and behaviour will develop. The leader has established their approach to nursing research and has also taken an active position on the importance of evidence‐based nursing. The leader can, when this level is reached, articulate and understand the differences and similarities in research in general and can assess how nursing research increases the quality of nursing care through evidence‐based nursing.

### 3.6 The outcome space: The individual path—How to make vision and reality become a working entity around evidence‐based nursing

The conceptions presented in the outcome space can be understood to depict a development from the lowest level, where the daily work as FLNM involves putting out fires and trying to get the clinical work done from one day to another, as seen in Figure 1. At this level, there is confusion about the differences between medical and nursing research, for example; also, the understanding of evidence‐based nursing is vague in many ways. Another issue, of time constraints seen as an obstacle, is presented at the next level of the outcome space. Time as an obstacle can be either subjective or real. Moving further up in the hierarchic path of the outcome space, some insight might appear, though there can still be obstacles to understanding and getting a grip on nursing research and how it can lead the way to evidence‐based nursing. However, once they have reached this level in the outcome space, FLNM's understand and sometimes even embrace the importance of their own contribution to evidence‐based nursing on the ward, an opportunity to be able to work supportive. When reaching the highest level of the outcome space, the category of description could be understood as a way of organizing the clinical work at the ward in a reflected and articulated way so that nursing will be evidence‐based. The fundamental link for the outcome space is clarification of the individual path the FLNM has to embrace when taking the lead towards achieving evidence‐based nursing. This was articulated as: “The individual path—how to make vision and reality become a working entity to support evidence‐based nursing”.

## 4 DISCUSSION

The aim was to explore first line nurse managers’ experiences of opportunities and obstacles to support evidence‐based nursing. The results show a complex situation regarding the responsibility and the FLNM's view was unclear concerning their own leadership in this responsibility. Confusion regarding how evidence‐based nursing could be understood was presented, together with the participants’ own varying thoughts regarding how they could facilitate evidence‐based nursing, both can be understood as obstacles. To support something to which one has an unreflective or shallow approach is of course difficult and sometimes even impossible. In this climate, medical research becomes the norm. And, furthermore, there is a risk that nurses do not value research conducted to improve nursing care, in line with what other studies report, for example (Bohman et al., 2013). A recent study (Berthelsen & Hølge‐Hazelton, 2017) underlines that what its authors call “nursing research culture” is present when evidence‐based nursing is the current approach. The authors describe a climate built on research use with acceptance from colleagues and management, together with the support and facilitation as factors needed (Berthelsen & Hølge‐Hazelton, 2017). Parts of these attributes were described in our study as desirable and seen as opportunities, but the attributes could not being understand as present.

The participants in this study described a working day strained by duties. On a daily basis, emergency situations arose and the participants had to avert situations that could affect the work on the ward, like handling absence due to illness among the nursing staff or scheduling for busy periods of time. The contextual prerequisites, for example, where the FLNM's felt that management put production as their highest priority, played a significant role when formulating the FLNM's own priorities. When FLNMs’ do not feel in control of their own position and work situation, it is almost impossible to achieve a supportive approach to evidence‐based nursing. In a quantitative study (Johansson, Sandahl, & Hasson, 2013), the results showed that when the FLNM feels in control of the job situation they cope better with high‐demand job situations.

The issue of time and the approach towards time, can be understood in different ways in this study. The participants declared that they lacked the time to support the nurses towards evidence‐based nursing and if only they “had more time” they would give the support. In a recent study from Denmark (Bundgaard, Sörensen, & Delmar, 2016), the authors argue for an approach shaped by how one can use the time one has, instead of trying to get or wish for more time. They underline the importance of spending time well and not just letting it pass. Time is an entity that needs to be processed, rather than an obstacle. Health care today often presents a view of time that is based on time as a subject rather than an approach, as if time was something touchable and concrete instead of something we have to handle or use. This can lead to the experience of being at the mercy of time and a feeling of hopelessness when time is never sufficient. Time can also be considered as an accepted excuse; an obstacle, for choosing between different tasks; one prioritizes the most important task and if one does not have time for a certain task it is because that task is not important enough. The way the manager sets priorities affects how nurses value and use their own time, how they set their own priorities in nursing care and how much time they spend with their patients, for example (Gunawan & Aungsuroch, 2017). If the FLNM is a person who “never has time” or always is busy the nurses working under this FLNM are likely to adopt the same attitude towards their patients (Perreira & Berta, 2016).

In this study, some of the participants expressed awareness of their own importance in the process of nursing care becoming evidence‐based. They did not have a plan or a strategy for how to achieve evidence‐based nursing, but they saw themselves as “bearer of the culture” and understood the importance of their position as an opportunity. In a concept analysis on managerial competence with regard to the FLNMs’ role (Gunawan & Aungsuroch, 2017), their model shows how different attributes of the FLNM and different activities, such as planning, organizing and leading, have a direct impact on nurses’ performance and nurse and patient outcomes. This is supported by another study (Boström, Rudman, Ehrenberg, Gustavsson, & Wallin, 2013), who also argue for a plan or a strategy for how to support newly graduated nurses in working evidence‐based in nursing care. To be a supportive FLNM is not something that can be “done by itself”. It takes courage, competence, will and planning to achieve (Boström et al., 2013; Richter et al., 2016; Salmela, Koskinen, & Eriksson, 2017; Xiao, Yilan, & Qingsong, 2015).

The gaining of insight into their importance in supporting the nurses with strategies and tools sets FLNMs’ on an individual path towards supporting evidence‐based nursing. Notable was that nursing theories as a theoretical foundation in nursing were not discussed at all among the participants in this study (Alligood & Marriner‐Tomey, 2010). This is a common position, at least in the Nordic countries, even though knowledge of nursing theory can enhance clinical competence (Levy‐Malmberg & Hilli, 2014). The FLNM needs to embrace and understand the importance of nursing theory to be able to use this kind of research as a way to improve clinical competence. To enable implementation of nursing theory and work with the theories as an opportunity in clinical work, the FLNM has to ensure that there is time for reflection and discussion among the nurses. However, this will often be a challenging task due to the demand for production from the organization.

The outcome space draws a picture of an internal path, a journey FLNMs’ need to clarify for themselves. The individual FLNM need to be aware of the opportunities and obstacles there is, to be able to support evidence‐based nursing. Awareness and knowledge, as well as own beliefs that leadership has an important impact on how nursing becomes evidence‐based, are of importance, together with a vision and a plan to “get there”; these together can be understood as the internal relation, showing how active leadership influences evidence‐based nursing, a result in accordance with recent research (Fleiszer, Semenic, Ritchie, Richer, & Denis, 2016).

### 4.1 Study limitations and strengths

The trigger question used in the interviews was based on the results from another study (Lindberg et al., 2012) to have a starting point for the discussion. We found this strategy to work out fine since the participants in the different focus group settings discussed engaged. On the other hand, the result from that article could have influenced data analysis. This was handled through frequent discussions in the research team and through our results being presented and discussed in two different seminar settings. The eventual influence of the presented study on the participants’ expressions, was considered small since the result from that study was summarized and only served to initiate the discussion between the participants.

Focus group interviews are becoming more used for data collection in studies using phenomenography as method for data analysis (Arveklev, Berg, Wigert, Morrison‐Helme, & Lepp, 2018; Loan Minh et al., 2016). The choice of using focus groups in this study was based on the apprehension that if a researcher asks about evidence‐based nursing, the participant (as a FLNM) could experience this as a questioning of the FLNM's intention to work with that particular question. The settings of the focus groups varied between three and four participants to one setting with six participants, where the setting of three or four participants in the focus group was found more well focused and oriented to the phenomenon in interest. In this study, the setting helped the participants to share their conceptions in an advanced and reflective way (Freeman, 2006; Överlien, Aronsson, & Hydén, 2005). There would be no principal obstacle to using any kind of data in a phenomenographic approach as long as it supports the participants in sharing their experiences, conceptions and understandings in qualitative variations (Kroksmark, 2007; Marton & Booth, 2009). The participants’ varying background regarding experience, age and sex, contributed to variation in this study.

## 5 CONCLUSION

This study highlights the importance of FLNMs’ own approach towards evidence‐based nursing as a prerequisite for being able to support nurses in clinical nursing care. First line nurse managers need to reflect on their preunderstanding of their leadership role and function in relation to evidence‐based nursing. The organization surrounding the FLNMs’, for example, the hospital management and department management, needs to have an articulated strategy to support the FLNMs’ in achieving evidence‐based nursing, this to become an opportunity for the FLNMs’. The FLNMs’ need to have knowledge, courage and a strategic plan to succeed in creating the climate where evidence‐based nursing becomes a part of the daily clinical work.

The frustration about evidence‐based nursing expressed by the FLNM's in this study, concerns the everyday workload and time constraint as obstacles that affects FLNMs’ ability to support nurses to work evidence‐based in nursing care. Of even more importance is the confusion of evidence‐based nursing, what evidence‐based nursing stands for and how it can be understood among the FLNMs’ as well as the lack of orientation regarding how nursing theory could contribute to the care given. However, the individual FLNM who sees an active approach to nursing research and evidence‐based nursing as an opportunity, displays an awareness of the importance of their role or function when supporting nurses to work evidence‐based. To be aware of both opportunities and obstacles towards evidence‐based nursing can help the FLNM to realize how her or his approach will guide her or his path to become a working entity supporting nurses in clinical work.

## CONFLICTS OF INTEREST

None to declare.

## AUTHOR CONTRIBUTION

All authors are responsible for the reported research, and worked together with design, analysis and interpretation of data. All authors also approved to resubmit the paper to your journal. All authors agreed on the final version and meet at least one of the following criteria recommended by the ICMJE (https://www.icmje.org/recommondations/)

- substantial contributions to conception and design, acquisition of data or analysis and interpretation of data
- drafting the article or critically revising it for important intellectual content
